# Intensive care after vascular surgery: systematic review

**DOI:** 10.1093/bjs/znaf172

**Published:** 2025-08-09

**Authors:** Kitty H F Wong, Alex K Murigu, Gianluca Buongiovanni, Ronelle Mouton, Robert J Hinchliffe

**Affiliations:** Department of Vascular Surgery, Bristol Medical School, University of Bristol, Bristol, UK; Department of Vascular Surgery, Bristol Medical School, University of Bristol, Bristol, UK; Postgraduate School of Vascular Surgery, Università Degli Studi di Milano, Milan, Italy; Department of Anaesthesia, North Bristol NHS Trust, Bristol, UK; Department of Vascular Surgery, Bristol Medical School, University of Bristol, Bristol, UK; Department of Vascular Surgery, North Bristol NHS Trust, Bristol, UK

## Abstract

**Background:**

The optimal use of ICU resources in patients undergoing vascular surgery is unclear. The aim of this systematic review was to evaluate the impact of ICU admission on clinical outcomes and costs after elective and emergency vascular surgery.

**Methods:**

MEDLINE, Embase, the Cochrane Library, Cochrane Collaboration Central Register of Controlled Trials (CENTRAL), and trial registry databases were searched in July 2024. Studies comparing ICU care with intermediary or ward-based care for major vascular surgery patients were included.

**Results:**

Thirteen studies (11 elective only and 2 including emergencies) involving 157 932 patients met the inclusion criteria. ICU admission was associated with higher adjusted 30-day or in-hospital mortality (OR 4.14 (95% c.i. 1.65 to 10.41), *P* = 0.003; Grading of Recommendations Assessment, Development, and Evaluation (GRADE) certainty: moderate). Unadjusted analyses found ICU admission was associated with increased major adverse cardiovascular events (risk ratio (RR) 1.45 (95% c.i. 1.04 to 2.01), *P* = 0.030; GRADE certainty: very low), acute kidney injury (RR 1.98 (95% c.i. 1.49 to 2.63), *P* < 0.001; GRADE certainty: moderate), dialysis (RR 1.76 (95% c.i. 1.13 to 2.74), *P* = 0.010; GRADE certainty: low), readmission (RR 1.93 (95% c.i. 1.20 to 3.12), *P* = 0.007; GRADE certainty: moderate), and major bleeding (RR 1.37 (95% c.i. 1.03 to 1.81), *P* = 0.030; GRADE certainty: moderate). Respiratory failure requiring mechanical ventilation and infection were higher in patients admitted to ICU compared with ward-based care specifically. Hospital-associated costs were higher for ICU admission across all procedures.

**Conclusion:**

No clear clinical benefit was associated with ICU admission after vascular surgery. This may be due to residual confounding and insufficient risk stratification.

## Teaser

There is a lack of clinical guidelines to inform patient selection for intensive care unit (ICU) admission following vascular surgery. This systematic review found that ICU admission was associated with increased adjusted perioperative mortality compared to ward-based or intermediary levels of care, as well as other unadjusted adverse outcomes, and had increased costs. Both patient and hospital-level factors play a role in decisionmaking for ICU admission. The results suggest current risk-stratification tools are insufficient and future research accounting for measured and unmeasured confounders are needed to identify patient groups who would benefit most from ICU admission.

## Introduction

Patients undergoing vascular surgery are one of the frailest populations with high rates of perioperative mortality and morbidity^1^. This is especially true for the rising number of vascular patients presenting for urgent and emergency operations, where there is limited opportunity for medical optimization. Consequently, these procedures are associated with significant costs and resource utilization, including ICU admission to support postoperative recovery and prevent complications through high-level nursing care, invasive monitoring, and organ support^2^. Although the proportion of vascular patients requiring ICU admission after surgery has declined over time, through centralization of services and improvements in perioperative care, observational studies have reported an absolute increase in the number of vascular ICU admissions against a backdrop of increasing acute patient volume^2–4^.

The availability of intensive care beds is limited worldwide. In the UK, some 347–400 urgent operations per month were cancelled due to lack of critical care bed capacity in 2018–2019, contributing to missed time-dependent care targets in vascular surgery^5^. Meticulous selection of patients for ICU admission is necessary to ensure appropriate allocation of finite resources to those that need it most. However, current national and international guidelines for triaging vascular patients to intensive care are lacking. While there is general consensus that patients undergoing abdominal aortic aneurysm (AAA) repair or emergencies (for example aneurysm rupture, aortic dissection, and vascular trauma) should go to ICU after surgery^6–8^, recommendations for other procedures (for example, lower limb surgery for peripheral arterial disease) are not available, despite a high perioperative mortality of up to 6–8%^1^. In contrast, a mortality risk of ≥5% is often considered the threshold for ICU admission in general surgery as recommended by the National Emergency Laparotomy Audit (NELA)^9,10^. Applying the same risk thresholds to vascular surgery is not practicable and the definition of a ‘high-risk’ vascular patient who would benefit most from ICU admission is lacking. Intensive care resources are not always reliably allocated to the highest-risk patients to prevent complications and adverse outcomes^11,12^. The aim of this systematic review was to assess the clinical impact and costs of ICU admission after elective or emergency vascular surgery.

## Methods

A systematic review was conducted in accordance with the PRISMA guidelines^13^. MEDLINE, Embase, The Cochrane Library database, Cochrane Collaboration Central Register of Controlled Trials (CENTRAL) and trial registry databases, ClinicalTrials.gov, and the International Clinical Trials Registry Platform (ICTRP) were searched from inception to 30 July 2024 to identify studies reporting outcomes for patients admitted to ICU *versus* other levels of care after elective or emergency vascular surgery. No restrictions were made regarding publication type or language. Grey literature was not searched (*Table S1*). The study was registered in the International Prospective Register of Systematic Reviews (PROSPERO) on 27 August 2024 (https://www.crd.york.ac.uk/prospero/display_record.php?ID=CRD42024582644).

### Study selection

Any studies comparing direct ICU admission (that is the immediate destination after surgery) with other levels of care (intermediary care (high-dependency care or other level 2 care configurations) or ward-based care) for adults aged >18 years undergoing elective or emergency vascular surgery for AAA, carotid artery stenosis, or peripheral arterial disease (including lower limb revascularization with open surgical bypass or endovascular intervention and major amputations) were eligible for inclusion. Definitions of ICU admission varied across studies and were based on what the original authors or data sources designated as intensive or critical care. These classifications relied on institutional labels, postoperative location, or administrative coding, rather than admissions limited to patients with an ‘absolute indication’ for intensive care admission, such as the need for mechanical ventilation or advanced organ support.

Single-arm studies, non-comparative observational studies, and studies comparing outcomes after non-vascular surgery procedures were excluded. Intracranial, cardiac, false, or pseudo aneurysms, aneurysms secondary to dissection or trauma, procedures involving endovascular aneurysm sealing devices, and venous procedures were also excluded. Systematic reviews, traditional reviews, case reports, letters, editorials, and animal studies were excluded. Where incomplete or missing data were present, attempts were made to contact authors for further clarification. References of relevant studies were hand-searched for eligible studies. Two reviewers (A.K.M. and G.B.) independently screened titles, abstracts, and full texts of potentially eligible studies. Any disputes were resolved by discussion or by a third reviewer (K.H.F.W.). Screening was carried out using the Rayyan web app (http://rayyan.qcri.org/)^14^.

### Data extraction

Two reviewers (A.K.M. and G.B.) independently extracted pre-specified data from included studies, including study characteristics (title, publication year, publication journal, first author, language, study design, study duration, study location and setting, number of participants in study, funding sources, and inclusion and exclusion criteria) and patient demographics (age, sex, smoking status, BMI, co-morbidities, ASA grade, and surgical procedure received by participants).

### Outcomes

The primary outcome was 30-day or in-hospital mortality. Secondary outcomes included major adverse cardiovascular events (MACE; composite outcome of myocardial infarction, stroke, or cardiovascular death), major adverse limb events (MALE; composite outcome of limb ischaemia, major amputation, or ipsilateral limb revascularization), postoperative complications (including renal, respiratory, and infection), 30-day or in-hospital major bleeding, reintervention within index admission, length of hospital stay, readmission rates, hospital-associated costs, and factors influencing ICU admission on univariable or multivariable analysis. Outcomes reported by two or more studies were eligible for meta-analysis. Where possible, both unadjusted and adjusted results were extracted. Subgroup analyses were planned for all outcomes, according to the level of postoperative care received and according to the surgical intervention received, as well as according to whether patients were admitted as elective or emergency cases.

### Quality assessment

Study quality was assessed by two independent reviewers (A.K.M. and G.B.) using the risk of bias in non-randomized studies of interventions (ROBINS-I) tool^15^. The robvis web app was used to generate risk-of-bias plots^16^. The certainty of evidence was assessed using the Grading of Recommendations Assessment, Development, and Evaluation (GRADE) approach for each outcome using GRADEpro GDT software (https://www.gradepro.org)^17^. GRADE classifies the quality of evidence from included studies into ‘high’, ‘moderate’, ‘low’, and ‘very low’ quality.

### Statistical analysis

Data from included studies were pooled for meta-analysis using Review Manager software (Cochrane Collaboration)^18^. Analysis of unadjusted dichotomous variables was performed and risk ratios (RR) are reported. Continuous variables are reported using the mean difference (MD). For studies reporting median values, these values were converted into mean and standard deviation values using the method of Wan *et al*.^19^. Random-effects models were used due to low data quality. For studies reporting adjusted results, data were pooled using generic inverse variance and are presented as OR. *P* < 0.050 was considered statistically significant for all outcomes. Interstudy heterogeneity was examined using the *I*^2^ statistic and reporting bias assessments were planned using funnel plots if more than ten studies reported the same outcome. Sensitivity analyses were performed by excluding studies with a high risk of bias.

## Results

**Fig. 1:**
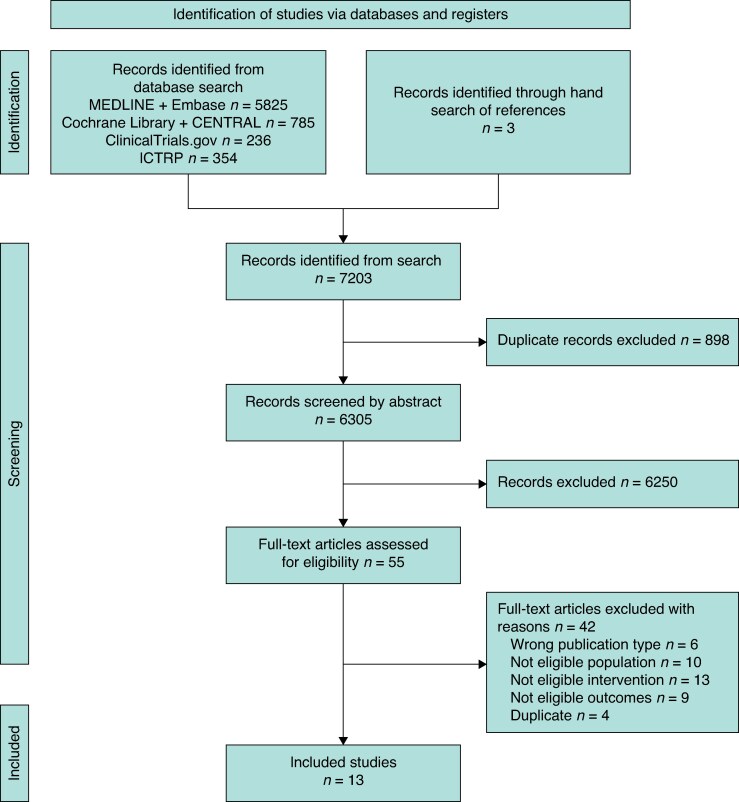
PRISMA flow diagram showing literature search used to obtain studies comparing the impact of intensive care admission with other levels of care for patients after elective or emergency vascular surgery CENTRAL, Cochrane Collaboration Central Register of Controlled Trials; ICTRP, International Clinical Trials Registry Platform.

A total of 6967 publications were obtained from the search and 104 articles were assessed as full texts. Of these, 13 papers (157 932 patients) were included^20–32^ (*Fig. 1*).

### Study characteristics

Full study characteristics and baseline demographics are presented within *Table 1* and *Table S2*. All included studies were observational in design (1 prospective and 12 retrospective), with no randomized trials. Follow-up ranged from 17 to 192 months. The mean age ranged from 66 to 74 years and the majority of included patients were male. Patients undergoing vascular surgery had significant co-morbidities, including diabetes mellitus, ischaemic heart disease, and renal failure, which were generally higher in patients admitted to ICU compared with those admitted to other levels of care. Four studies examined patients who underwent open AAA repair^21,22,25,26^, two studied elective endovascular aneurysm repair (EVAR)^30,32^, two studied carotid endarterectomy (CEA)^20,24^, and one focused on lower extremity bypass (LEB)^31^. One study evaluated both open AAA repair and CEA^23^, while another assessed both open AAA repair and EVAR^28^ . Two studies did not explicitly define the vascular procedures being investigated^27,29^. Eleven studies described elective vascular operations, one included both elective and emergency surgeries^27^, and one included emergency surgery only^29^.

**Table 1:** Study characteristics of observational studies comparing the impact of intensive care admission with other levels of care for patients after elective or emergency vascular surgery

Study (first author)	Type of study	Publication year	Total number of vascular patients	Surgical procedure received	Intervention arm	Number	Comparator arm	Number
L. Kraiss^20^	Retrospective observational	1995	196	CEA	ICU	178	General ward	18
D. Bertges^21^	Retrospective observational	2000	314	Open AAA repair	ICU	245	General ward	69
D. Lawlor^22^	Retrospective observational	2004	230	Open AAA repair	ICU	23	General ward	207
M. Norwood^23^	Retrospective observational	2004	122	Open AAA repair or CEA	ICU/HDU	52	General ward	70
D. Angel^24^	Prospective observational	2004	104	CEA	ICU	84	Intermediary level of care	20
C. Callaghan^25^	Retrospective observational	2005	178	Open AAA repair	ICU	26	Intermediary level of care	152
M. Cleary^26^	Retrospective observational	2006	104	Open AAA repair	ICU	57	Intermediary level of care	47
T. Engelbert^27^	Retrospective observational	2014	2505	Any vascular procedure	ICU	71	Unspecified	2434
H. Wunsch^28^	Retrospective observational	2016	98 665	Open AAA repair or EVAR	ICU	32 888	Unspecified	65 777
M. Gillies^29^	Retrospective observational	2017	5528	Any vascular procedure	ICU		Unspecified	
C. Hicks^30^	Retrospective observational	2018	8359	EVAR	ICU	4791	General ward	3568
H. Alshaikh^31^	Retrospective observational	2019	6010	LEB	ICU	2772	General ward	3238
T. Cheng^32^	Retrospective observational	2021	35 617	EVAR	ICU	5443	Unspecified	30 174

CEA, carotid endarterectomy; AAA, abdominal aortic aneurysm; HDU, high-dependency unit; EVAR, endovascular aneurysm repair; LEB, lower extremity bypass.

Three studies (386 patients) compared direct postoperative ICU admission with intermediary-level care^24–26^, while five studies (15 109 patients) compared ICU admission with ward-based care^20–22,30,31^. Four studies (142 315 patients) compared ICU care with any other levels of care, but did not specify what this entailed^27–29,32^. One study (122 patients) compared ICU or high-dependency care with ward-based care and was described separately^23^.

### Quality assessment

**Fig. 2:**
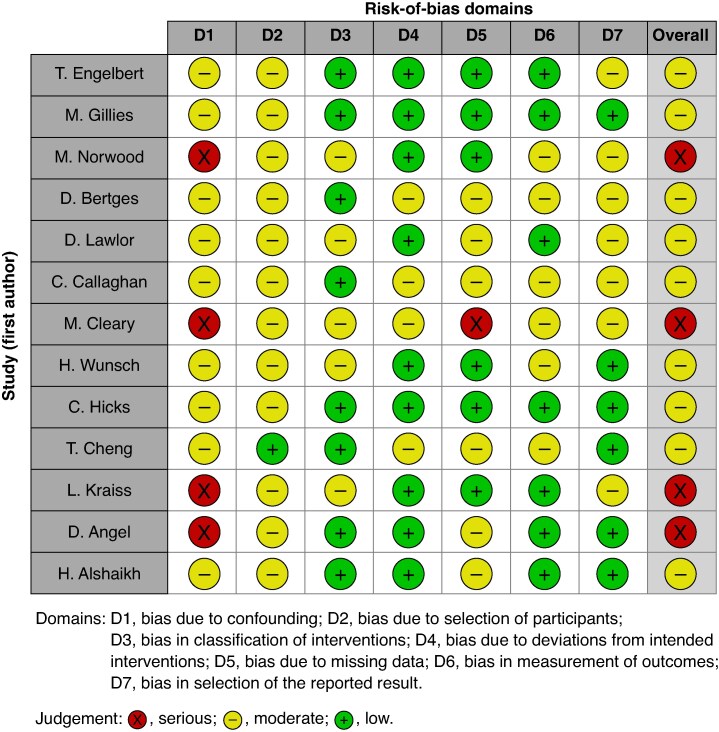
Risk-of-bias assessments using the ROBINS-I tool for observational studies comparing the impact of intensive care admission with other levels of care for patients after elective or emergency vascular surgery ROBINS-I, risk of bias in non-randomized studies of interventions.

The quality of studies was low, with four having a ‘serious’ risk of bias^20,23,24,26^ and the remaining nine having a ‘moderate’ risk (*Fig. 2*)^21,22,25,27–32^. The low quality was mostly attributed to the risk of confounding and selection bias due to the observational nature of the studies. Publication bias could not be assessed as none of the outcomes was reported by at least ten studies. The GRADE certainty assessments for all outcomes are presented in *Table 2*, ranging from ‘very low’ to ‘high’.

**Table 2:** Summary-of-findings table presenting certainty assessments for primary and secondary outcomes of observational studies comparing intensive care admission with other levels of care after elective or emergency vascular surgery

Outcomes	Anticipated absolute effects* (95% c.i.)		Relative effect (95% c.i.)	No. of participants (no. of studies)	Surgical procedure received (no. of studies)	Certainty of the evidence (GRADE)	Comments
	Risk with postoperative admission to other levels of care	Risk with postoperative admission to ICU					
Thirty-day or in-hospital mortality (unadjusted results)	18 per 1000	26 per 1000 (14–49)	RR 1.44 (0.76,2.73)	51 008 (8)^20–22,25,26,30–32^	Open AAA repair (4)^21,22^ ^,25^ ^,26^ EVAR (2)^30,32^ CEA (1)^20^ LEB (1)^31^	Very low	*P* = 0.260, *I*^2^ = 49% Sensitivity analysis: RR 1.80 (95% c.i. 0.90,3.63), *P* = 0.100
Thirty-day or in-hospital mortality (adjusted results)			OR 4.14 (1.65,10.41)	104 193 (2)^28,29^	Any vascular procedure (1)^29^ Open AAA repair (1)^28^ EVAR (1)^28^	Moderate	*P* = 0.003, *I*^2^ = 98% Not enough data for sensitivity analysis
MACE	9 per 1000	13 per 1000 (10–18)	RR 1.45 (1.04,2.01)	50 704 (7)^20,21,24,26,30–32^	Open AAA repair (2)^21,26^ EVAR (2)^30,32^ CEA (2)^20,24^ LEB (1)^31^	Very low	*P* = 0.030, *I*^2^ = 40% Sensitivity analysis: RR 1.55 (95% c.i 1.12,2.15), *P* = 0.009
Heart failure and arrhythmia	8 per 1000	11 per 1000 (7–18)	RR 1.31 (0.79,2.16)	36 743 (7)^20–22,24–26,32^	Open AAA repair (4)^21,22,25,26^ EVAR (1)^32^ CEA (2)^20^ ^,24^	Very low	*P* = 0.290, *I*^2^ = 76% Sensitivity analysis: RR 1.83 (95% c.i. 1.03,3.25), *P* = 0.040
MALE	9 per 1000	9 per 1000 (7–11)	RR 0.96 (0.77,1.20)	41 731 (3)^26,31,32^	Open AAA repair (1)^26^ EVAR (1)^32^ LEB (1)^31^	Low	*P* = 0.720, *I*^2^ = 0% Sensitivity analysis: RR 0.97 (95% c.i. 0.78,1.21), *P* = 0.770
AKI	23 per 1000	46 per 1000 (35,61)	RR 1.98 (1.49,2.63)	6188 (2)^25,31^	Open AAA repair (1)^25^ LEB (1)^31^	Moderate	*P* < 0.001, *I*^2^ = 0% Not enough data for sensitivity analysis
Renal failure requiring dialysis	16 per 1000	28 per 1000 (18,44)	RR 1.76 (1.13,2.74)	15 017 (5)^21,22,26,30,31^	Open AAA repair (3)^21,22,26^ EVAR (1)^30^ LEB (1)^31^	Low	*P* = 0.010, *I*^2^ = 40% Sensitivity analysis: RR 1.90 (95% c.i. 1.14,3.16), *P* = 0.010
Respiratory complications	13 per 1000	15 per 1000 (8,28)	RR 1.12 (0.60,2.11)	36 213 (4)^21,25,26,32^	Open AAA repair (3)^21,25,26^ EVAR (1)^32^	Very low	*P* = 0.720, *I*^2^ = 83% Sensitivity analysis: RR 1.41 (95% c.i. 0.72,2.73), *P* = 0.310
Respiratory failure requiring mechanical ventilation	13 per 1000	36 per 1000 (13,101)	RR 2.83 (1.00,8.01)	9185 (5)^21,22,25,26,30^	Open AAA repair (4)^21,22,25,26^ EVAR (1)^30^	Very low	*P* = 0.050, *I*^2^ = 67% Sensitivity analysis: RR 4.25 (95% c.i. 1.61,11.18), *P* = 0.003
Infection	46 per 1000	49 per 1000 (32,76)	RR 1.08 (0.70,1.67)	8955 (4)^21,25,26,30^	Open AAA repair (3)^21,25,26^ EVAR (1)^30^	Very low	*P* = 0.710, *I*^2^ = 58% Sensitivity analysis: RR 1.11 (95% c.i. 0.60,2.08), *P* = 0.740
Surgical-site infection	3 per 1000	2 per 1000 (1,4)	RR 0.75 (0.45,1.23)	41 909 (4)^25,26,31,32^	Open AAA repair (2)^25,26^ EVAR (1)^32^ LEB (1)^31^	Low	*P* = 0.250, *I*^2^ = 0% Sensitivity analysis: RR 0.74 (95% c.i. 0.45,1.24), *P* = 0.250
Readmission	93 per 1000	180 per 1000 (112,290)	RR 1.93 (1.20,3.12)	2819 (2)^21,27^	Any vascular procedure (1)^27^ Open AAA repair (1)^21^	Moderate	*P* = 0.007, *I*^2^ = 0% Not enough data for sensitivity analysis
Thirty-day or in-hospital major bleeding	20 per 1000	28 per 1000 (21,37)	RR 1.37 (1.03,1.81)	9059 (5)^21,24–26,30^	Open AAA repair (3)^21,25,26^ EVAR (1)^30^ CEA (1)^24^	Moderate	*P* = 0.030, *I*^2^ = 0% Sensitivity analysis: RR 1.38 (95% c.i. 1.04,1.84), *P* = 0.030
Reintervention within index admission	16 per 1000	20 per 1000 (14,31)	RR 1.24 (0.83,1.87)	42 453 (6)^21,22,24,25,31,32^	Open AAA repair (3)^21,22,25^ EVAR (1)^32^ CEA (1)^24^ LEB (1)^31^	Very low	*P* = 0.30, *I*^2^ = 50% Sensitivity analysis: RR 1.28 (95% c.i. 0.82,1.99), *P* = 0.270
Length of hospital stay		MD 0.59 (0.53,1.71)	–	42 105 (5)^20,24,25,31,32^	Open AAA repair (1)^25^ EVAR (1)^32^ CEA (2)^20,24^ LEB (1)^31^	Very low	*P* = 0.30, *I*^2^ = 100% Sensitivity analysis: MD 0.10 (95% c.i. −0.10,0.30), *P* = 0.330

^*^The risk in the intervention group (and its 95% confidence interval) is based on the assumed risk in the comparison group and the relative effect of the intervention (and its confidence interval). GRADE Working Group grades of evidence: high certainty, very confident that the true effect lies close to that of the estimate of the effect; moderate certainty, moderately confident in the effect estimate (the true effect is likely to be close to the estimate of the effect, but there is a possibility that it is substantially different); low certainty, confidence in the effect estimate is limited (the true effect may be substantially different from the estimate of the effect); and very low certainty, very little confidence in the effect estimate (the true effect is likely to be substantially different from the estimate of the effect). GRADE, Grading of Recommendations Assessment, Development, and Evaluation; RR, risk ratio; AAA, abdominal aortic aneurysm; EVAR, endovascular aneurysm repair; CEA, carotid endarterectomy; LEB, lower extremity bypass; MACE, major adverse cardiovascular events; MALE, major adverse limb events; AKI, acute kidney injury; MD, mean difference.

### Primary outcome: 30-day or in-hospital mortality

Eight studies (51 008 patients; all elective cases) presented unadjusted outcomes and were pooled for meta-analysis^20–22,25,26,30–32^. There was no significant difference in 30-day or in-hospital mortality between patients admitted to ICU and those admitted to other levels of care (RR 1.44 (95% c.i. 0.76 to 2.73), *I*^2^ = 49%, *P* = 0.260; GRADE certainty: very low). Subgroup analyses did not find a significant difference based on the level of postoperative care or intervention received (*Table S3*).

**Fig. 3:**
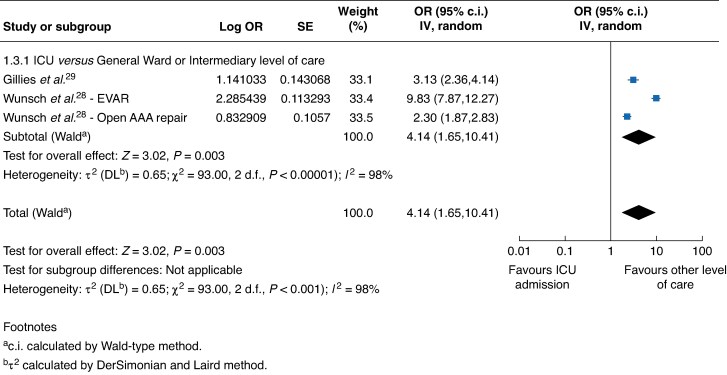
Forest plot showing adjusted 30-day or in-hospital mortality OR for observational studies comparing admission to intensive care with other levels of care for patients after elective or emergency vascular surgery, grouped by level of postoperative care received SE, standard error; IV, inverse variance; EVAR, endovascular aneurysm repair; AAA, abdominal aortic aneurysm.

Two studies (104 193 patients; one elective and one emergency) reported adjusted mortality rates using multivariable regression analysis and propensity matching to account for patient- and hospital-level variables (*Fig. 3*)^28,29^. Pooled analysis found ICU admission was associated with a higher 30-day or in-hospital mortality compared with other levels of care (OR 4.14 (95% c.i. 1.65 to 10.41), *I*^2^ = 98%, *P* = 0.003; GRADE certainty: moderate). One study (129 227 patients) found that this difference was more pronounced in patients undergoing elective EVAR compared with open AAA repair on subgroup analysis^28^. Gillies *et al*.^29^ (5528 patients) found indirect ICU admission (admission to ICU after a period of care in a non-ICU environment after surgery) was associated with a two-fold increase in 30-day mortality compared with direct ICU admission (admission to ICU directly from theatre) in emergency vascular surgery.

### Secondary outcomes

#### MACE

Seven studies (50 704 elective patients) observed higher unadjusted MACE rates in patients admitted to ICU compared with other levels of care (RR 1.45 (95% c.i. 1.04 to 2.01), *I*^2^ = 40%, *P* = 0.030; GRADE certainty: very low)^20,21,24,26,30–32^. This remained significant on subgroup analysis comparing ICU with ward-based care specifically.

#### MALE

Three studies (41 731 elective patients) did not find a significant difference in unadjusted MALE across intervention arms (RR 0.96 (95% c.i. 0.77 to 1.20), *I*^2^ = 0%, *P* = 0.720; GRADE certainty: low)^26,31,32^.

#### Renal complications

Two studies (6188 elective patients) found an association between higher unadjusted rates of acute kidney injury (AKI) for patients admitted to ICU *versus* other levels of care after surgery (RR 1.98 (95% c.i. 1.49 to 2.63), *I*^2^ = 0%, *P* < 0.001; GRADE certainty: moderate)^25,31^.

Five studies (15 017 elective patients) found ICU admission was associated with a higher unadjusted risk of renal failure requiring dialysis support compared with other levels of care (RR 1.76 (95% c.i. 1.13 to 2.74), *I*^2^ = 40%, *P* = 0.010; GRADE certainty: low)^21,22,26,30,31^. This remained significant on subgroup comparison between ICU and ward-based care.

#### Respiratory complications

Four studies (36 213 elective patients) found no statistically significant difference in unadjusted respiratory complications after surgery across intervention arms (RR 1.12 (95% c.i. 0.60 to 2.11), *I*^2^ = 83%, *P* = 0.720; GRADE certainty: very low)^21,25,26,32^. Only one study compared ICU with ward-based care, which found a significantly higher rate of respiratory complications in patients admitted to ICU^21^.

Five studies (9185 elective patients) found no significant difference in unadjusted respiratory failure requiring mechanical ventilation for patients admitted to ICU *versus* other levels of care (RR 2.83 (95% c.i. 1.00 to 8.01), *I*^2^ = 67%, *P* = 0.050; GRADE certainty: very low)^21,22,25,26,30^. However, this became significant on sensitivity analysis excluding one study with a serious risk of bias (RR 4.25 (95% c.i. 1.61 to 11.18), *I*^2^ = 55%, *P* = 0.003; GRADE certainty: low).

#### Infection

Four studies (8955 elective patients) found no significant difference in unadjusted infection rates across groups (RR 1.08 (95% c.i. 0.70 to 1.67), *I*^2^ = 58%, *P* = 0.710; GRADE certainty: very low)^21,25,26,30^. On subgroup analysis, patients admitted to ICU had a higher rate of infection compared with patients receiving ward-based care specifically.

#### Thirty-day or in-hospital major bleeding

Five studies (9059 elective patients) found higher unadjusted 30-day or in-hospital major bleeding for patients admitted to ICU compared with other levels of care (RR 1.37 (95% c.i. 1.03 to 1.81), *I*^2^ = 0%, *P* = 0.030; GRADE certainty: moderate)^21,24–26^  ^,30^. This difference persisted for subgroup analysis comparing ICU admission with ward-based care, but not when comparing ICU with intermediary care.

#### Reintervention within index admission

Meta-analysis of six studies (42 453 elective patients) did not find a difference in unadjusted reintervention rates between patients admitted to ICU *versus* other levels of care after surgery (RR 1.24 (95% c.i. 0.83 to 1.87), *I*^2^ = 50%, *P* = 0.300; GRADE certainty: very low)^21,22,24,25,31,32^. However, ICU admission was associated with higher reintervention rates after open AAA repair on subgroup analysis. One study also reported a higher reintervention rate in patients undergoing EVAR who were admitted to ICU after surgery^32^.

#### Readmission

Two studies (2819 patients; one elective and one elective and emergency) found a significant difference in unadjusted readmission rates between ICU and other levels of care after surgery (RR 1.93 (95% c.i. 1.20 to 3.12), *I*^2^ = 0%, *P* = 0.007; GRADE certainty: moderate)^21^  ^,27^. This was mainly driven by higher rates of readmission reported by Engelbert *et al*.^27^ (2505 patients undergoing elective or emergency vascular surgery), although they noted that this effect was attenuated on multivariable analysis accounting for demographic and clinical variables.

#### Length of hospital stay

Five studies (42 105 patients) showed no significant difference in unadjusted length of stay between groups (MD 0.59 (95% c.i. −0.53 to 1.71), *I*^2^ = 100%, *P* = 0.300; GRADE certainty: very low)^20,24,25,31,32^. One study found greater lengths of stay in patients admitted to ICU after EVAR compared with those who were admitted to other levels of care^32^.

#### Hospital-associated costs

Six studies (113 648 patients) reported unadjusted hospital-associated costs, but meta-analysis was not possible due to reporting heterogeneity^20,21,26,28,30,31^. All studies reported higher costs for ICU admission compared with other levels of care, with this being observed across various vascular procedures, including EVAR^28,30^, open AAA repair^21,26,28^, CEA^20^, and LEB^31^.

#### Factors influencing ICU admission

Seven studies (50 686 patients) examined factors influencing ICU admission through either univariable^21,24,25,32^ or multivariable^30,31^ regression analysis (*Table S4*). Six studies included demographic data, such as age, sex, and co-morbidities, in their model. Three considered procedure-specific data, such as aneurysm morphology, contrast use, and clamp time and position. Two studies included hospital-level factors. Both patient-level characteristics (including co-morbidities, increased transfusion need, and longer operating room time) and hospital-level characteristics (including geographical location, whether or not a hospital is a teaching hospital, and ICU bed availability) were identified as significant predictors for ICU admission. However, the same factors were not always consistently significant across studies.

## Discussion

This systematic review identified 13 observational studies assessing the outcomes of postoperative ICU admission for adult patients undergoing elective and emergency vascular surgery. On meta-analysis of low-quality observational data, ICU admission was associated with increased adjusted mortality compared with other levels of care, as well as unadjusted adverse perioperative outcomes, including MACE, AKI, dialysis requirement, readmission, and major bleeding. Compared with ward-based care, ICU admission was associated with higher unadjusted rates of respiratory failure requiring mechanical ventilation and infection. ICU admission was consistently more costly than other levels of care and decision-making was influenced by patient- and hospital-level factors.

The results of this study echo prior literature, which was unable to demonstrate which patients benefit from ICU admission after surgery across surgical specialties, calling for prompt reflection on the risk-stratification process and added value of intensive care after major vascular surgery^33^. ICU-based interventions, such as organ support or invasive monitoring^34,35^, have been shown to improve patient outcomes, but are often not required for planned ICU admissions directly from theatre^36^. Rather, the benefit of this complex intervention may actually derive from enhanced nursing, early recognition, and management of postoperative complications in an environment with additional resources that are difficult to access in ward-based settings^37^. Crucially, planned admissions directly from theatre could prevent unplanned escalation to ICU or a high-dependency unit, which is consistently associated with poor outcomes^29^. The higher incidence of unadjusted perioperative complications in patients admitted to ICU in this review may therefore reflect appropriate selection of higher-risk patients (who invariably have more risk factors for poor outcomes) and attempts to prevent failure to rescue, as demonstrated by higher rates of reintervention, mechanical ventilation, and dialysis compared with ward-based care. Conversely, US-based studies for patients undergoing elective infrainguinal bypass for claudication and standard infrarenal EVAR, usually considered a ‘low risk’ group^1^, did not observe significant differences in postoperative complications between patients routinely admitted to ICU and those admitted to the ward^30,31^.

However, despite adjusting for patient- and hospital-level variables, perioperative mortality rates remained significantly higher in patients admitted to ICU after surgery. This was probably due to residual confounding, which is difficult to adequately account for in observational studies (for example intraoperative events, individual unit configurations and cultures, and whether ICU admission was planned or unplanned)^28,29^. While both studies reporting adjusted mortality rates accounted for common confounders, such as age, sex, and co-morbidity, many of the factors significantly influencing ICU admission identified in other observational studies (*Table S4*), such as intraoperative blood loss, specific procedural steps, medications, and surgeon experience, were not included as model covariates. Additionally, external factors, such as ICU capacity and hospital-level factors, which have been shown to significantly influence ICU admission, were not considered^30,31,38^. Similar increases in perioperative mortality and morbidity were attenuated on advanced statistical analysis accounting for unobserved confounding in the multinational second Sprint National Anaesthesia Project (SNAP2 EpiCCS) study across surgical specialties, but, nonetheless, no clear survival benefit was observed with ICU admission^38^. There were no randomized trials comparing intensive care *versus* other levels of care identified in this review nor across other surgical specialties to provide ‘gold standard’ evidence, possibly due to ethical and practical difficulties, with slow recruitment and availability of intensive care beds precluding randomization^39,40^.

More importantly, these results suggest a lack of a robust risk-stratification process to select patients who would benefit most from costly and finite intensive care resources, particularly for patients without an ‘absolute indication’ for ICU admission. This is recognized as a UK national research priority by the National Confidential Enquiry into Perioperative Deaths (NCEPOD), the Getting it Right First Time (GIRFT) initiative, and the James Lind Alliance^41–46^. This is especially essential in vascular surgery, which involves one of the frailest, most underserved, and high-risk patient populations among all surgical specialties^1,11,29^. Current national and international vascular guidelines from the European Society of Vascular Surgery, the Society of Vascular Surgery, and the Vascular Society of Great Britain and Ireland are limited to recommendations of ICU admission for patients undergoing open AAA repair or emergency aortic surgery only^6–8^. No formal recommendations are made for patients undergoing CEA, EVAR, or LEB^6,47,48^, despite high perioperative mortality rates that exceed commonly accepted risk thresholds for ICU admission^9^. General surgical risk-prediction models often have significant variability and are poorly calibrated to vascular patients who tend to have a poor functional baseline and multimorbidity, and there is a lack of widely accepted and validated vascular-specific risk scores to inform prognostication^49^. This has led to widespread variation in the use of intensive care for vascular patients, with suboptimal allocation of resources manifesting in both lack of intensive care provision for patients who suffer adverse events (failure to rescue and a high rate of unplanned ICU admissions, which is associated with worse outcomes) and use of intensive care services for patients who perhaps did not require them^41^. In turn, this could cascade into other undesirable consequences, including surgical cancellations (which are strongly predicted by a lack of intensive care beds)^50^ and delays in treatment for vascular patients who tend to be on a time-dependent treatment pathway. In response, some quality-improvement programmes in the UK have highlighted the need to reduce unnecessary ICU admissions for patients undergoing CEA and EVAR, but, again, specific guidance on patient selection is lacking^41,51^.

While the decision to admit to ICU may be based on a number of patient factors, important external factors, such as hospital policy, intensive care bed capacity, operating volume, and geographical location, have been shown to play a role^30,31,38,52,53^. Decision-making surrounding ICU admission is complex and often lacks transparent protocolized care, with varying importance given to different factors by different clinicians^52^. This raises the question of whether there are potential biases in clinical decision-making that systematically disadvantage certain patient subpopulations and if this could be reduced with standardization of services and equity in provision of perioperative care^38,54^. On the other hand, greater systematic use of intensive care without considering individual patient risk has not shown improvement in survival, is costly, and may take away resources from those who need it most^28,30,31^. Qualitative research studies could shed further light on how healthcare professionals approach decision-making in ICU admissions and explore influencing factors. Future research should focus on developing vascular-specific risk-prediction tools and consider novel methodologies (such as advanced causal inference and quasi-experimental studies) to account for unobserved confounders in the absence of randomized evidence, in line with the National Institute for Health and Care Excellence (NICE) real-world evidence framework^55^. However, challenges remain in widespread adoption of such studies due to the availability of routinely collected data and it may be necessary to adapt patient registries going forward to enable robust analysis of contemporary observational data.

This review provides a comprehensive and contemporary summary of the evidence for intensive care use in vascular surgery. However, there are several limitations. All included studies were observational, with no randomized trials, and data quality was generally low. Aside from mortality, only unadjusted outcomes were available without adjustment for variables. The results are therefore prone to selection bias and measured and unmeasured confounding. Additionally, there was limited clarity regarding the type of care delivered, which may introduce bias given the evolving nature of intermediary care configurations, increasing specialist recovery wards with enhanced nursing, critical care outreach teams, and other quality-improvement interventions introduced over time. The proportions of planned and unplanned ICU admissions were unclear, probably owing to the retrospective nature of these studies, precluding further insights into decision-making and allocation of intensive care resources. It was also unclear how many patients had an ‘absolute indication’ for ICU admission (such as requiring mechanical ventilation) *versus* those admitted for enhanced observation only, but who theoretically could be managed in a normal ward-based setting. Finally, data were insufficient to enable complete subgroup analysis according to procedure for each outcome and to stratify patients according to elective or emergency admission.

Careful selection of patients for intensive care is necessary to ensure limited resources are directed towards those who will benefit most. In this systematic review of low-quality data, no clear clinical advantages were observed with direct ICU admission after vascular surgery and it was associated with increased costs. While this may represent appropriate admission of the highest-risk patients, measured and unmeasured biases and confounding likely contribute and highlight the lack of robust risk-stratification processes for intensive care admission. Future research should utilize randomization or advanced statistical methodology to inform critical care patient selection balancing patient safety with optimal resource allocation.

## Supplementary Material

znaf172_Supplementary_Data

## Data Availability

The authors confirm that the data supporting the findings of this study are available within the article and its *Supplementary material*.
