# Supplementary material for: Intensive care after vascular surgery: systematic review
Source: Br J Surg. 2025 Aug 9;112(8):znaf172. doi: 10.1093/bjs/znaf172 (PMC12342744; doi:10.1093/bjs/znaf172)
Supplement: znaf172_Supplementary_Data [file znaf172_supplementary_data.docx]

**Impact of intensive care on patients undergoing vascular surgery: a systematic review**

**Authors:** Kitty H.F. Wong^a*^, Alex K. Murigu^a*^, Gianluca Buongiovanni^b^, Ronelle Mouton^c^ Robert J. Hinchliffe^a,d^

a. Department of Vascular Surgery, Bristol Medical School, University of Bristol, Bristol, UK.

b. Postgraduate School of Vascular Surgery, Università Degli Studi di Milano, Milan, Italy

c. Department of Anaesthesia, North Bristol NHS Trust, Bristol, UK

d. North Bristol NHS Trust, Bristol, UK.

* Joint first authors

**Corresponding Author:** Miss Kitty HF Wong, 2nd Floor Learning & Research Building, Southmead Hospital, Bristol BS10 5NB, UK.

Email: [kitty.wong@bristol.ac.uk](mailto:kitty.wong@bristol.ac.uk)

ORCiD: 0000-0002-1498-0316

Twitter / X: @kittywonghf

**Supplementary Materials - Index**

| **Supplementary Appendixes** |  |
| --- | --- |
| Changes to methodology following PROSPERO registration | *page 3* |
| **Supplementary Figures and Tables** |  |
| Supplementary Table S1. Full search strategy performed to obtain studies comparing the impact of intensive care admission to other levels of care for patients after elective or emergency vascular surgery | *page 4* |
| Supplementary Table S2. Full study characteristics and baseline demographic details of included observational studies comparing the impact of intensive care admission to other levels of care for patients after elective or emergency vascular surgery.  Supplementary Table S3. Full summary of findings table presenting certainty assessments for primary and secondary outcomes of observational studies comparing intensive care admission to other levels of care following elective or emergency vascular surgery.  Supplementary Table S4. Full details of factors influencing admission to intensive care of included observational studies comparing the impact of intensive care admission to other levels of care for patients after elective or emergency vascular surgery. | *page 24*  *Page 27*  *Page 36* |

**Supplementary Appendixes**

Changes to methodology following PROSPERO registration

| **Change made** | **Rationale** |
| --- | --- |
| Mortality measure changed from hazard ratio to odds ratio | Only aggregate data was reported in the included studies for mortality using adjusted odds ratio measures. It was not possible to extract data required to provide a pooled hazard ratio. |
| Length of stay changed from hazard ratio to mean difference | Included papers reported aggregate results with a mixture of mean (standard deviation) and median (interquartile range) values. Based on the data available it was not possible to determine a hazard ratio, but MD could be calculated by converting median to mean. |
| Risk ratio used instead of odds ratio for dichotomous outcomes | Some outcomes (e.g. respiratory complications and infections) had a high event rate. Therefore RR was used across outcomes to avoid overestimation. |

**Supplementary Table S1.** Full search strategy performed to obtain studies comparing the impact of intensive care admission to other levels of care for patients after elective or emergency vascular surgery

MeSH terms used for searching the MEDLINE and EMBASE databases via the Ovid platform. Last search date: 30 July 2024

| 1 | exp vascular surgery/ | 912 761 |
| --- | --- | --- |
| 2 | exp aneurysm/ | 319 981 |
| 3 | Aortic Aneurysm, Abdominal/ | 34 613 |
| 4 | (aneurysm* adj3 (abdom* or aort* or infrarenal* or infra-renal*)).tw. | 105 187 |
| 5 | AAA.ti,ab. | 40 980 |
| 6 | (aneurys* adj3 (extracranial or extra-cranial or peripheral or peripher* or limb* or popliteal* or carotid* or subclavian* or axillar* or brachial* or arter* or iliac* or femoral* or femor* or crural or pedal)).ti,ab. | 72 738 |
| 7 | 2 or 3 or 4 or 5 or 6 | 386 910 |
| 8 | (aneurys* adj3 (venous or vein* or ven*)).ti,ab. | 16 386 |
| 9 | (aneurys* adj4 (false or trauma* or rupture* or thorac* or thoracoabdom* or thoraco-abdom* or spontan* or renal* or juxtarenal* or juxta-renal* or juxta renal* or paraerenal* or para-renal* or para renal* or suprarenal* or supra renal* or supra-renal* or short neck* or short-neck* or shortneck* or hostile neck)).ti,ab. | 90 839 |
| 10 | (pseudoaneurysm* or pseudo-aneurysm* or trauma* or rupture*).ti,ab. | 1 389 863 |
| 11 | (dissection* or dissect* or intramural* or IMH or TBAD or AAD).ti,ab. | 584 035 |
| 12 | (intracranial or intra-cranial* or berry* or cerebral or cerebr* or intracerebral or intra-cerebral or brain* or subarachnoid or perinatal or sub-arachnoid or extradural or extra-dural or subdural or sub-dural or basilar* or vertebral or cerebellar).ti,ab. | 4 565 840 |
| 13 | (coronar* or cardi* or ventric* or atrial or Kawasaki*).ti,ab. | 4 990 699 |
| 14 | (Behcet* or CTD* or connective tissue* or Marfan* or Ehlers-Danlos or Ehlers* or arteritis or autoimmune).ti,ab. | 766 737 |
| 15 | (Open repair or OSR or open or FEVAR or BEVAR or ChEVAR or REVAR or ((fenestrated or fenestrat* or branch* or chimney or complex or thora* or thoracoabdom* or thoraco-abdom* or ruptur*) adj3 (EVAR or endovasc* or endopros* or endograft* or stent* or repair*))).ti,ab. | 1 565 179 |
| 16 | 8 or 9 or 10 or 11 or 12 or 13 or 14 or 15 | 12 495 643 |
| 17 | 7 not 16 | 104 738 |
| 18 | (Repair* or EVAR or endovasc* or endother* or stent* or graft* or endopros* or endograft* or reconstr* or interven* or surg* or operat* or proce* or treat*).ti,ab. | 30 400 208 |
| 19 | 17 and 18 | 52 296 |
| 20 | ((peripheral or percutaneous or intervention) adj3 endovascular).ti,ab. | 12 193 |
| 21 | angioplasty/ or angioplasty, balloon/ or angioplasty, balloon.mp. or angioplasty, balloon, laser-assisted/ or angioplasty, laser/ [mp=ti, ab, hw, tn, ot, dm, mf, dv, kf, fx, dq, bt, nm, ox, px, rx, ui, sy, ux, mx] | 123 123 |
| 22 | (angioplast* or (stent* adj4 (bare or metal or balloon or expand*)) or graft* or endograft* or endoprosthe* or PTA or DEB or DCB or PEB or PCB or paclitaxel or PTX or (drug adj4 (elut* or cover* or stent*)) or atherectom*).ti,ab. | 1 305 689 |
| 23 | ((limb? or leg? or foot or feet or lower extremit* or ((above or below) adj2 (knee? or ankle?))) adj3 (arter* or isch?emi* or lesion? or recanali* or revascular* or segment or thrombo* or surgery or intervention)).ti,ab. | 113 006 |
| 24 | 20 or 21 or 22 or 23 | 1 448 114 |
| 25 | (bypass$ or by-pass$ or revascularization$ or re-vascularization$ or revascu$ or shunt$ or graft$ or grafting$).ti,ab. | 1 521 269 |
| 26 | exp Vascular Grafting/ | 252 450 |
| 27 | 25 or 26 | 1 602 971 |
| 28 | exp Ischemia/ or Intermittent Claudication/ or Peripheral Arterial Disease/ or exp Arterial Occlusive Diseases/ or Atherosclerosis/ or (peripheral adj3 (arter* or vascular*) adj3 (disease or isch?emi* or segment or occlus* or patency or reocclus* or re-occlus* or steno* or restenos* or revascular* or recanali* or obstruct* or lesio* or block* or harden* or stiffen* or obliter* or intervention or surgery)).ti,ab,kf. | 1 769 944 |
| 29 | (ischemia$ or ischaemia$ or claudication$ or (peripheral$ adj3 arter$ adj3 disease$) or (arter$ adj3 (obstructive$ or occlusive$) adj3 disease$)).mp. | 1 026 181 |
| 30 | 28 or 29 | 2 139 517 |
| 31 | Femoral Artery/ or Popliteal Artery/ or (femorofemoral$ or femoral-femoral or (femoropopliteal$ or (femoral$ adj2 popliteal$) or fem-pop) or (infrainguinal$ or (infra$ adj3 inguinal$) or infra-inguinal$)).mp. | 95 838 |
| 32 | exp Lower Extremity/ or ((lower$ adj3 (extremit$ or limb$)) or (membrum adj3 inferius$)).mp. | 906 804 |
| 33 | 31 or 32 | 979 734 |
| 34 | 27 and 30 and 33 | 32 480 |
| 35 | 24 and 33 | 126 952 |
| 36 | (AKA or BKA or (Amputat* adj3 (limb* or leg* or lower extremity* or knee* or ankle* or above knee or below knee or major))).ti,ab. | 52 464 |
| 37 | exp Amputation, Surgical/ | 85 186 |
| 38 | 36 or 37 | 110 910 |
| 39 | Carotid Artery/ or carotid*.ti,ab. | 344 896 |
| 40 | (Endarterectomy or endarterectomy* or stent* or mesh* or repair* or surg* or angioplast* or CEA or CAS).ti,ab. | 6 916 367 |
| 41 | 39 and 40 | 106 011 |
| 42 | 1 or 19 or 34 or 35 or 38 or 41 | 1 157 813 |
| 43 | exp Critical Care/ or exp Intensive Care Units/ or (ICU or ITU or HDU or CCU or SICU or ward*).ti. | 1 311 813 |
| 44 | ((care or unit* or ward*) adj3 (intens* or critic* or high depend* or high-depend* or high care or high-care or depend* or step down or step-down or step-up or step up or transitional care or medium care or intermediate care or intermediary care)).ti. | 179 019 |
| 45 | 43 or 44 | 1 337 328 |
| 46 | 42 and 45 | 52 319 |
| 47 | (child* or neonat* or p#diatric* or infant* or p#d*).ti,ab. | 6 423 311 |
| 48 | 46 not 47 | 46 185 |
| 49 | limit 48 to humans | 43 903 |
| 50 | (coron* or coronary or neuro* or $neuro$ or heart or ventric* or atria* or atrium or intracranial* or intra-cranial* or cerebral* or intracerebral* or cerebr* or brain or subarach* or sub-arach* or $dural$ or dissect* or hepatic or liver or cardiovascular or upper limb or upper extremity or orthop#dic or pelvic floor or burn* or $cardia* or mitral* or bicuspid* or tricuspid* or $cardiu*).ti,ab. | 15 790 242 |
| 51 | Vena Cava/ or Pulmonary Artery/ or pulmonary arter*.ti,ab. or pulmona*.ti. or arterial line*.ti,ab. or peripheral arterial catheter*.ti,ab. or peripheral arterial line*.ti,ab. or arterial cannulation.ti. or arterial cannula.ti. or tunnelled line*.ti,ab. or (fistul* or venous thrombosis or central line or PICC or pulmonary embol* or IVC or vena cav* or portal vein or porto* or cannula* or cannulation or venepuncture* or venipuncture* or ((vein* or venous or central or IV or intravenous or intra-venous) adj4 (portal or porto* or line* or thrombos* or embol* or cathet* or cannula* or cannulation or access or venepuncture* or venipuncture*))).ti,ab. [mp=ti, ab, hw, tn, ot, dm, mf, dv, kf, fx, dq, bt, nm, ox, px, rx, ui, sy, ux, mx] | 1 673 757 |
| 52 | 50 or 51 | 16 834 936 |
| 53 | 49 not 52 | 10 421 |
| 54 | (systematic or structured or evidence or trials or studies).ti. and ((review or overview or look or examination or update* or summary).ti. or review.pt.) | 796 682 |
| 55 | meta-analysis.pt. or (meta-analys* or meta analys* or metaanalys* or meta synth* or meta-synth* or metasynth*).ti,ab,kf,hw. | 846 734 |
| 56 | ((systematic or meta) adj2 (analys* or review)).ti,kf. or ((systematic* or quantitativ* or methodologic*) adj5 (review* or overview*)).ti,ab,kf,sh. or (quantitativ$ adj5 synthesis$).ti,ab,kf,hw. | 1 155 377 |
| 57 | (integrative research review* or research integration).tw. or scoping review?.ti,kf. or (review.ti,kf,pt. and (trials as topic or studies as topic).hw.) or (evidence adj3 review*).ti,ab,kf. | 365 003 |
| 58 | review.pt. and ((medline or medlars or embase or pubmed or scisearch or psychinfo or psycinfo or psychlit or psyclit or cinahl or electronic database* or bibliographic database* or computeri#ed database* or online database* or pooling or pooled or mantel haenszel or peto or dersimonian or der simonian or fixed effect or ((hand adj2 search*) or (manual* adj2 search*))).tw,hw. or (retraction of publication or retracted publication).pt.) | 510 453 |
| 59 | (case report or case study).ti. | 866 652 |
| 60 | 54 or 55 or 56 or 57 or 58 or 59 | 2 730 406 |
| 61 | 53 not 60 | 9 526 |
| 62 | (mortalit$ or death$ or crude death$ or surviv$ or fatal$ or pain or $pathy or $pathic or $algia or nocicep$ or migrator$ or radiat$ or chronic pain$ or morbidit$ or co-morbidit$ or comorbidit$ or $infect$ or re-infect$ or reinfect$).ti,ab. | 14 989 848 |
| 63 | (cost$ or cost-benefit$ or cost benefit$ or cost-effectiv$ or cost effectiv$ or expendit$ or rate$ or incidenc$ or prevalen$ or life expect$ or life year$ or life-expect$ or life-year$ or ((quality or disability or health) adj3 (adjust$ or life or year$ or tim$)) or QALY$ or DALY$ or HALY$).ti,ab. | 14 561 836 |
| 64 | ((major adj3 (cardi$ or limb or advers$ or bleed$ or transfu$ or h#emorr$ or blood or loss or amput$)) or re-operat$ or reoperat$ or revascul$ or re-vascul$ or reinterv$ or reinterv$ or revis$ or ((repeat$ adj2 (operat$ or interv$ or proced$ or surg$ or stent$ or revascul$ or re-vascul$)) or correct$)).ti,ab. | 2 966 342 |
| 65 | ((number or length or time$ or patient or post-op$ or postop$ or operat$ or duration or continuity or $long$ or chronic or requir$ or utili$ or necess$) adj3 (discharg$ or stay or handoff$ or hand-off$ or transfe$ or admission$ or readmission$ or re-admission$ or $hospitali#at$ or treatmen$ or care$ or complic$ or concer$ or intens$ or critic$ or ICU or CCU or SICU or $ventil$ or $intub$ or inotrop$ or $press$ or recov$ or condition$ or transfus$ or oxygen or HDU or high car$ or high-car$ or depend$ or ward or supp$)).ti,ab. | 5 055 650 |
| 66 | (risk$ or odd$ or hazard$ or probabi$ or likelih$ or outcom$ or differentia$ or analy$ or endpoint$ or end-point$ or time-frame or time frame or time-point or timepoint or variabl$ or measur$ or assess$ or efficac$ or effectiv$ or scor$ or scal$ or grad$ or dropout$ or drop-out$ or impact$ or thirty or 30 day or 30-day or ((patient or health$) adj2 (centr$ or cente$ or report$ or outcom$ or saf$))).ti,ab. | 42 072 407 |
| 67 | (frail$ or ((base$ or baseline or base line or base-line or function$ or statu$) adj2 (chang$ or normal or declin$ or decreas$ or ris$ or increas$ or drop$ or reduc$ or optimi$ or detrior$ or improv$))).ti,ab. | 1 424 571 |
| 68 | 62 or 63 or 64 or 65 or 66 or 67 | 49 557 203 |
| 69 | 61 and 68 | 6 053 |
| 70 | limit 69 to yr="2015 -Current" | 3 563 |
| 71 | remove duplicates from 70 | 3 474 |
| 72 | limit 69 to yr="1860 - 2014" | 2 488 |
| 73 | remove duplicates from 72 | 2 351 |
| 74 | 71 or 73 | 5 825 |

MeSH terms used for searching the Cochrane Library database and Cochrane Collaboration Central Register of Controlled Trials (CENTRAL). Last search date: 30 July 2024

| #1 | MeSH descriptor: [Vascular Surgical Procedures] explode all trees | 22 398 |
| --- | --- | --- |
| #2 | MeSH descriptor: [Aneurysm] explode all trees | 2 257 |
| #3 | MeSH descriptor: [Aortic Aneurysm, Abdominal] explode all trees | 890 |
| #4 | (aneurysm* near/3 (abdom* or aort* or infrarenal* or infra-renal*)) | 2 399 |
| #5 | (aneurys* near/3 (extracranial or extra-cranial or peripheral or peripher* or limb* or popliteal* or carotid* or subclavian* or axillar* or brachial* or arter* or iliac* or femoral* or femor* or crural or pedal)) | 1 017 |
| #6 | #2 or #3 or #4 or #5 | 3 918 |
| #7 | (aneurys* near/3 (venous or vein* or ven*)) | 117 |
| #8 | (aneurys* near/4 (false or trauma* or rupture* or thorac* or thoracoabdom* or thoraco-abdom* or spontan* or renal* or juxtarenal* or juxta-renal* or juxta renal* or paraerenal* or para-renal* or para renal* or suprarenal* or supra renal* or supra-renal* or short neck* or short-neck* or shortneck* or hostile neck)) | 1 903 |
| #9 | (pseudoaneurysm* or pseudo-aneurysm* or trauma* or rupture*) | 53 516 |
| #10 | (dissection* or dissect* or intramural* or IMH or TBAD or AAD) | 15 275 |
| #11 | (intracranial or intra-cranial* or berry* or cerebral or cerebr* or intracerebral or intra-cerebral or brain* or subarachnoid or perinatal or sub-arachnoid or extradural or extra-dural or subdural or sub-dural or basilar* or vertebral or cerebellar) | 162 889 |
| #12 | (coronar* or cardi* or ventric* or atrial or Kawasaki*) | 267 237 |
| #13 | (Behcet* or CTD* or connective tissue* or Marfan* or Ehlers-Danlos or Ehlers* or arteritis or autoimmune) | 17 897 |
| #14 | (Open repair or OSR or open or FEVAR or BEVAR or ChEVAR or REVAR or ((fenestrated or fenestrat* or branch* or chimney or complex or thora* or thoracoabdom* or thoraco-abdom* or ruptur*) near/3 (EVAR or endovasc* or endopros* or endograft* or stent* or repair*))) | 191 135 |
| #15 | #7 or #8 or #9 or #10 or #11 or #12 or #13 or #14 | 607 777 |
| #16 | #6 not #15 | 666 |
| #17 | (Repair* or EVAR or endovasc* or endother* or stent* or graft* or endopros* or endograft* or reconstr* or interven* or surg* or operat* or proce* or treat*) | 1 734 506 |
| #18 | #16 and #17 | 611 |
| #19 | ((peripheral or percutaneous or intervention) near/3 endovascular) | 508 |
| #20 | angioplasty or balloon* or laser-assisted* | 19 512 |
| #21 | (angioplast* or (stent* near/4 (bare or metal or balloon or expand*)) or graft* or endograft* or endoprosthe* or PTA or DEB or DCB or PEB or PCB or paclitaxel or PTX or (drug near/4 (elut* or cover* or stent*)) or atherectom*) | 69 207 |
| #22 | ((limb* or leg* or foot or feet or lower extremit* or ((above or below) near/2 (knee? or ankle?))) near/3 (arter* or isch?emi* or lesion* or recanali* or revascular* or segment or thrombo* or surgery or intervention)) | 22 897 |
| #23 | #19 or #20 or #21 or #22 | 97 377 |
| #24 | bypass* or by-pass* or revascularization** or re-vascularization* or revascu* or shunt* or graft* or grafting* | 69 298 |
| #25 | MeSH descriptor: [Vascular Grafting] explode all trees | 8 383 |
| #26 | #24 or #25 | 69 657 |
| #27 | MeSH descriptor: [Peripheral Arterial Disease] explode all trees | 2 689 |
| #28 | MeSH descriptor: [Intermittent Claudication] explode all trees | 1 192 |
| #29 | MeSH descriptor: [Atherosclerosis] explode all trees | 3 819 |
| #30 | MeSH descriptor: [Arterial Occlusive Diseases] explode all trees | 16 959 |
| #31 | (peripheral near/3 (arter* or vascular*) near/3 (disease or isch?emi* or segment or occlus* or patency or reocclus* or re-occlus* or steno* or restenos* or revascular* or recanali* or obstruct* or lesio* or block* or harden* or stiffen* or obliter* or intervention or surgery)) | 7 478 |
| #32 | (ischemia$ or ischaemia$ or claudication$ or (peripheral$ near/3 arter$ near/3 disease$) or (arter$ near/3 (obstructive$ or occlusive$) near/3 disease$)) | 32 189 |
| #33 | #27 or #28 or #29 or #30 or #31 or #32 | 47 757 |
| #34 | MeSH descriptor: [Femoral Artery] explode all trees | 1 376 |
| #35 | MeSH descriptor: [Popliteal Artery] explode all trees | 545 |
| #36 | femorofemoral* or femoral-femoral or (femoropopliteal* or (femoral* near/2 popliteal*) or fem-pop) or (infrainguinal* or (infra* near/3 inguinal*) or infra-inguinal*) | 1 625 |
| #37 | MeSH descriptor: [Lower Extremity] explode all trees | 10 235 |
| #38 | ((lower$ adj3 (extremit$ or limb$)) or (membrum adj3 inferius$)) | 416 |
| #39 | #34 or #35 or #36 or #37 or #38 | 12 904 |
| #40 | #26 and #33 and #39 | 1 192 |
| #41 | #23 and #39 | 3 690 |
| #42 | (AKA or BKA or (Amputat* near/3 (limb* or leg* or lower extremity* or knee* or ankle* or above knee or below knee or major))) | 2 715 |
| #43 | MeSH descriptor: [Amputation, Surgical] explode all trees | 688 |
| #44 | #42 or #43 | 2 972 |
| #45 | MeSH descriptor: [Carotid Arteries] explode all trees | 1 506 |
| #46 | (Endarterectomy or endarterectomy* or stent* or mesh* or repair* or surg* or angioplast* or CEA or CAS) | 432 051 |
| #47 | #45 and #46 | 455 |
| #48 | #1 or #18 or #40 or #41 or #44 or #47 | 28 089 |
| #49 | MeSH descriptor: [Critical Care] explode all trees | 3 059 |
| #50 | (ICU or ITU or HDU or CCU or SICU or ward*) | 45 770 |
| #51 | ((care or unit* or ward*) near/3 (intens* or critic* or high depend* or high-depend* or high care or high-care or depend* or step down or step-down or step-up or step up or transitional care or medium care or intermediate care or intermediary care)) | 371 844 |
| #52 | #50 or #51 | 390 225 |
| #53 | #48 and #52 | 6 690 |
| #54 | (child* or neonat* or p#diatric* or infant* or p#d*) | 276 239 |
| #55 | #53 not #54 | 6 099 |
| #56 | (coron* or coronary or neuro* or $neuro$ or heart or ventric* or atria* or atrium or intracranial* or intra-cranial* or cerebral* or intracerebral* or cerebr* or brain or subarach* or sub-arach* or $dural$ or dissect* or hepatic or liver or cardiovascular or upper limb or upper extremity or orthop#dic or pelvic floor or burn* or $cardia* or mitral* or bicuspid* or tricuspid* or $cardiu*) | 667 755 |
| #57 | MeSH descriptor: [Vena Cava, Inferior] explode all trees | 155 |
| #58 | MeSH descriptor: [Vena Cava, Superior] explode all trees | 97 |
| #59 | MeSH descriptor: [Pulmonary Artery] explode all trees | 640 |
| #60 | pulmonary arter*. or pulmona* or arterial line* or peripheral arterial catheter* or peripheral arterial line* or arterial cannulation or arterial cannula or tunnelled line* or (fistul* or venous thrombosis or central line or PICC or pulmonary embol* or IVC or vena cav* or portal vein or porto* or cannula* or cannulation or venepuncture* or venipuncture* or ((vein* or venous or central or IV or intravenous or intra-venous) near/4 (portal or porto* or line* or thrombos* or embol* or cathet* or cannula* or cannulation or access or venepuncture* or venipuncture*))) | 178 761 |
| #61 | #56 or #57 or #58 or #59 or #60 | 778 071 |
| #62 | #55 not #61 | 785 |

MeSH terms used for searching the trial registry databases ClinicalTrials.gov and International Clinical Trials Registry Platform (ICTRP). Last search date: 30 July 2024.

| Trial Registry | Condition | Intervention | Number of Hits |
| --- | --- | --- | --- |
| ClinicalTrials.gov | Aneurysm | Vascular Surgery | 94 |
| ClinicalTrials.gov | Carotid Artery Diseases | Vascular Surgery | 48 |
| ClinicalTrials.gov | Peripheral Artery Disease | Vascular Surgery | 94 |
| ICTRP | Aneurysm or Carotid or Peripheral Artery Disease | - | 354 |

# **Supplementary Table S2.** Full study characteristics and baseline demographic details of included observational studies comparing the impact of intensive care admission to other levels of care for patients after elective or emergency vascular surgery. ICU = intensive care unit; AAA = abdominal aortic aneurysm; HDU = high-dependency unit; EVAR = endovascular aneurysm repair; IQR = Interquartile Range; SDU = Surgical Day Unit; OIR = Overnight Intensive Recovery; BMI = Body Mass Index.

| **First Author** | **Study Duration (months)** | **Study Location** | **Funding Source** | **Age** | **Male Gender** | **Smoking** | **BMI** | **Hypertension** | **Hyperlipidaemia** | **Diabetes Mellitus** | **Ischaemic Heart Disease** | **Renal Failure** | **American Society of Anesthesiologist’s**  **(ASA) Class** | **Hospital-Associated Costs** |
| --- | --- | --- | --- | --- | --- | --- | --- | --- | --- | --- | --- | --- | --- | --- |
| L. Kraiss^20^ | 24 | University of Washington and Providence Medical Center (United States) | Not specified |  |  |  |  |  |  |  |  |  | ICU: 3.1 + 0.0  Ward: 2.8 + 0.1  p=.12 | Total Hospital Charges ICU: $11,140 ± $729  Ward: $5,861 ± $229  p=.02 |
| D. Bertges^21^ | 72 | University of Pittsburgh Medical Center  (United States) | Not specified | ICU: 71.4  Ward: 69.8 | ICU: 173 (70.6%)  Ward: 46 (66.7%) |  |  | ICU: 147 (60.0%)  Ward: 43 (62.3%) |  | ICU: 27 (11.0%)  Ward: 7 (10.1%) | ICU: 141 (57.6%)  Ward: 33 (47.8%) | ICU: 16 (6.5%)  Ward: 3 (4.3%) | ICU: 3  Ward: 3 | Mean Costs  ICU: $56,065  Ward: $32,919 |
| K.Lawlor^22^ | 26 | London Health Sciences Centre (Canada) | Not specified | Mean  ICU: 72  SDU: 72 |  |  |  |  |  |  |  |  |  |  |
| M. Norwood^23^ | 24 | Leicester General Hospital (United Kingdom) | Not specified |  |  |  |  |  |  |  |  |  |  |  |
| D. Angel^24^ | 17 | Royal Perth Hospital (Western Australia) | Not specified | ICU: 72  HDU: 66 | ICU: 59  HDU: 12 |  |  |  |  | ICU: 35%  HDU: 15% |  |  |  |  |
| C. Callaghan^25^ | 60 | Cambridge Vascular Unit  (United Kingdom) | Not specified | Median (IQR)  ICU: 71 (66-76)  OIR: 72 (66-77) | ICU: 22 (85%)  OIR: 134 (88%) | ICU: 8 (31%)  OIR: 57 (38%) |  | ICU: 18 (69%)  OIR: 73 (48%) |  | ICU: 3 (12%)  OIR: 12 (8%) | ICU: 11 (42%)  OIR: 23 (15%) | ICU: 8 (31%)  OIR: 39 (26%) | Frequencies 1:2:3:4  ICU: 0:5:18:2  OIR: 3:67:72:1 |  |
| M.Cleary^26^ | 66 | St. Vincent's University Hospital (Ireland) | Not specified | ICU: 71.5 +/- 0.95  HDU: 71.2 +/- 1.4 | ICU: 49  HDU: 36 |  |  |  |  |  |  |  |  | Overall Cost  ICU: €287,100  HDU: €236,350  Net saving: €50,750 |
| T. Engelbert^27^ | 53 | University of Wisconsin Hospitals and Clinics  (United States) | Commercial and Non-Commercial | Median 66 (IQR 18) | 1,521 (60.7%) |  |  |  |  |  |  |  |  |  |
| H. Wunsch^28^ | 60 | Sunnybrook Health Sciences Centre (Canada) | Non-Commercial |  |  |  |  |  |  |  |  |  |  | Mean Medicare Payments (SD) Low ICU Usage  EVAR: $72,200 (51,200) AAA $88,900 (137,000)   Moderate ICU Usage  EVAR: $83,800 (60,700) p<.001 AAA: $85,100 (96,100) p=.14   High ICU Usage EVAR: $80,300 (57,400) p<.001 AAA $126,600 (94,100) p=.03 |
| M. Gillies^29^ | 96 | Royal Infirmary of Edinburgh (Scotland) | Non-Commercial |  |  |  |  |  |  |  |  |  |  |  |
| C. Hicks^30^ | 68 | Johns Hopkins Medical Institutes and The Johns Hopkins Surgery Center for Outcomes Research, Johns Hopkins School of Medicine (United States) | Not specified | Median (IQR) ICU: 74 (68-80) No ICU: 73 (68-79) | ICU: 3,856 (80.5%)  No ICU: 2,931 (82.1%) | ICU Never: 2,093 (43.7%) Ever: 1,473 (30.7%) Current: 1,225 (25.6%)  No ICU Never: 1,381 (38.7%) Ever: 1,263 (35.4%) Current: 924 (25.9%) |  | ICU: 3,805 (79.4%)  No ICU: 2,718 (76.2%) |  | ICU: 972 (20.3%)  No ICU: 652 (18.3%) | ICU: 2,237 (46.7%)  No ICU: 1,513 (42.4%) | ICU: 46 (1.0%)  No ICU: 28 (0.8%) |  | Median (IQR) ICU: 27,029 (19,236-35,604)  No ICU: 25,091 (17,966-33,223) p<.001 |
| H. Alshaikh^31^ | 68 | Johns Hopkins Surgery Center for Outcomes Research (United States) | Not specified | Median (IQR) ICU: 66 (59-73)  Surgical Floor: 66 (59-73) | ICU: 1,889 (68.1%)  Surgical Floor: 2,175 (67.2%) | ICU  Never: 947 (34.2%)  Former: 814 (29.4%)  Current: 1,011 (36.5%)  Surgical Floor  Never: 1,101 (34.0%)  Former: 935 (28.9%)  Current: 1,202 (37.1%) |  | ICU: 2,207 (79.6%)  Surgical Floor: 2,582 (79.7%) | ICU: 1,648 (59.5%)  Surgical Floor: 1,938 (59.9%) | ICU: 1,023 (36.9%)  Surgical Floor: 1,069 (33.0%) | ICU: 1,309 (47.2%)  Surgical Floor: 1,323 (40.9%) | ICU: 44 (1.6%)  Surgical Floor: 35 (1.1%) |  | Median (IQR)  ICU: $13,273 ($10,136-$17,883)  Surgical Floor: $10,927 ($8,342-$14,523) |
| T.Cheng^32^ | 192 | Boston University School of Medicine (United States) | No funding | Routine ICU: 73.4 ± 8.4  Non-routine ICU: 73.4 ± 8.4 | Routine ICU: 4,381 (80.5%)  Non-routine ICU: 24,674 (81.8%) | Routine ICU  Never: 683 (12.6%)  Former: 3,014 (55.4%)  Current: 1,745 (32.1%)  Non-Routine ICU  Never: 3,848 (12.8%)  Former: 17,136 (56.8%)  Current: 9,167 (30.4%) | BMI ≥ 30kg/m²  Routine ICU: 1,679 (30.9%)  Non-routine ICU: 9,608 (32%) | Routine ICU: 4,516 (83%)  Non-routine ICU: 25,174 (83.5%) |  | Routine ICU: 1,116 (20.5%)  Non-routine ICU: 6,121 (20.3%) | Routine ICU: 1,611 (29.6%)  Non-routine ICU: 8,884 (29.5%) | Routine ICU: 79 (1.5%)  Non-routine ICU: 355 (1.2%) |  |  |

**Supplementary Table S3.** Full summary of findings table presenting certainty assessments for primary and secondary outcomes of observational studies comparing intensive care admission to other levels of care following elective or emergency vascular surgery.

| **Outcomes** | **Anticipated absolute effects^*^ (95% CI)** | | **Relative effect (95% CI)** | **№ of participants (studies)** | **Surgical procedure received (studies)** | **Certainty of the evidence (GRADE)** | **Comments** |
| --- | --- | --- | --- | --- | --- | --- | --- |
|  | **Risk with Postoperative Admission to Other Levels of Care** | **Risk with Postoperative Admission to ICU** |  |  |  |  |  |
| **30-Day or In-Hospital Mortality (Unadjusted Results)** | 18 per 1 000 | **26 per 1 000** (14 to 49) | **RR 1.44** (0.76 to 2.73) | 51 008  (8 studies)^20-22,25,26,30-32^ | Open AAA Repair (4)^21,22,25,26^  EVAR (2)^30,32^  CEA (1)^20^  LEB (1)^31^ | ⨁◯◯◯ Very low | p = 0.26, I² = 49%;  Sensitivity analysis: RR 1.80 (95% CI 0.90-3.63), p = 0.10 |
| ICU vs General Ward | 3 per 1 000 | **6 per 1 000** (2 to 20) | **RR 2.31** (0.72 to 7.34) | 15 109 (5 studies)^20-22,30,31^ | Open AAA Repair (2)^21,22^  EVAR (1)^30^  CEA (1)^20^  LEB (1)^31^ | ⨁◯◯◯ Very low | p = 0.16, I² = 61%;  Sensitivity analysis: RR 2.93 (95% CI 0.84-10.20), p = 0.090 |
| ICU vs Intermediary Level of Care | 30 per 1 000 | **27 per 1 000** (4 to 183) | **RR 0.91** (0.14 to 6.06) | 282 (2 studies)^25,26^ | Open AAA Repair (2)^25,26^ | ⨁◯◯◯ Very low | p = 0.92, I² = 44%;  Not enough data for sensitivity analysis |
| Open AAA Repair | 13 per 1 000 | **45 per 1 000** (5 to 398) | **RR 3.54** (0.40 to 31.49) | 826 (4 studies)^21,22,25,26^ |  | ⨁◯◯◯ Very low | p = 0.26, I² = 70%;  Sensitivity analysis: RR 8.57 (95% CI 1.14-64.52), p = 0.040 |
| EVAR | 4 per 1 000 | **4 per 1 000** (3 to 6) | **RR 1.06** (0.73 to 1.54) | 43 976 (2 studies)^30,32^ |  | ⨁⨁◯◯ Low | p = 0.77, I² = 0%;  Not enough data for sensitivity analysis |
| **30-Day or In-Hospital Mortality (Adjusted Results)** |  |  | **OR 4.14** (1.65 to 10.41) | 104 193 (2 studies)^28,29^ | Any Vascular Procedure (1)^29^  Open AAA Repair (1)^28^  EVAR (1)^28^ | ⨁⨁⨁◯ Moderate | p = 0.003, I² = 98%;  Not enough data for sensitivity analysis |
| **Major Adverse Cardiovascular Events (MACE)** | 9 per 1 000 | **13 per 1 000** (10 to 18) | **RR 1.45** (1.04 to 2.01) | 50 704 (7 studies)^20,21,24,26,30-32^ | Open AAA Repair (2)^21,26^  EVAR (2)^30,32^  CEA (2)^20,24^  LEB (1)^31^ | ⨁◯◯◯ Very low | p = 0.030, I² = 40%;  Sensitivity analysis: RR 1.55 (95% CI 1.12-2.15), p = 0.009 |
| ICU vs General Ward | 12 per 1 000 | **22 per 1 000** (17 to 29) | **RR 1.86** (1.43 to 2.41) | 14 879 (4 studies)^20,21,30,31^ | Open AAA Repair (1)^21^  EVAR (1)^30^  CEA (1)^20^  LEB (1)^31^ | ⨁⨁◯◯ Low | p < 0.001, I² = 0%;  Sensitivity analysis: RR 1.86 (95% CI 1.44-2.42), p < 0.001 |
| ICU vs Intermediary Level of Care | 75 per 1 000 | **29 per 1 000** (7 to 120) | **RR 0.39** (0.09 to 1.61) | 208 (2 studies)^24,26^ | Open AAA Repair (1)^26^  CEA (1)^24^ | ⨁◯◯◯ Very low | p = 0.19, I² = 0%;  Not enough data for sensitivity analysis |
| Open AAA Repair | 43 per 1 000 | **39 per 1 000** (3 to 501) | **RR 0.91** (0.07 to 11.63) | 418 (2 studies)^21,26^ |  | ⨁◯◯◯ Very low | p = 0.94, I² = 61%;  Not enough data for sensitivity analysis |
| EVAR | 9 per 1 000 | **14 per 1 000** (8 to 23) | **RR 1.52** (0.92 to 2.53) | 43 976 (2 studies)^30,32^ |  | ⨁◯◯◯ Very low | p = 0.10, I² = 83%;  Not enough data for sensitivity analysis |
| Carotid Endarterectomy | 0 per 1 000 | **0 per 1 000** (0 to 0) | **RR 1.05** (0.13 to 8.65) | 300 (2 studies)^20,24^ |  | ⨁◯◯◯ Very low | p = 0.97, I² = 0%;  Not enough data for sensitivity analysis |
| **Heart Failure and Arrhythmia** | 8 per 1 000 | **11 per 1 000** (7 to 18) | **RR 1.31** (0.79 to 2.16) | 36 743 (7 studies)^20-22,24-26,32^ | Open AAA Repair (4)^21,22,25,26^  EVAR (1)^32^  CEA (2)^20,24^ | ⨁◯◯◯ Very low | p = 0.29, I² = 76%;  Sensitivity analysis: RR 1.83 (95% CI 1.03-3.25), p = 0.040 |
| ICU vs General Ward | 129 per 1 000 | **216 per 1 000** (61 to 772) | **RR 1.67** (0.47 to 5.97) | 740 (3 studies)^20-22^ | Open AAA Repair (2)^21,22^  CEA (1)^20^ | ⨁◯◯◯ Very low | p = 0.43, I² = 77%;  Sensitivity analysis: RR 3.09 (95% CI 2.00-4.80), p < 0.001 |
| ICU vs Intermediary Level of Care | 242 per 1 000 | **227 per 1 000** (121 to 426) | **RR 0.94** (0.50 to 1.76) | 386 (3 studies)^24-26^ | Open AAA Repair (2)^25,26^  CEA (1)^24^ | ⨁◯◯◯ Very low | p = 0.84, I² = 58%;  Not enough data for sensitivity analysis |
| Open AAA Repair | 164 per 1 000 | **236 per 1 000** (94 to 594) | **RR 1.44** (0.57 to 3.62) | 826 (4 studies)^21,22,25,26^ |  | ⨁◯◯◯ Very low | p = 0.44, I² = 82%;  Sensitivity analysis: RR 2.14 (95% CI 1.04-4.37), p = 0.040 |
| Carotid Endarterectomy | 342 per 1 000 | **103 per 1 000** (3 to 1 000) | **RR 0.30** (0.01 to 9.93) | 300 (2 studies)^20,24^ |  | ⨁◯◯◯ Very low | p = 0.50, I² = 80%;  Not enough data for sensitivity analysis |
| **Major Adverse Limb Events (MALE)** | 9 per 1 000 | **9 per 1 000** (7 to 11) | **RR 0.96** (0.77 to 1.20) | 41 731 (3 studies)^26,31,32^ | Open AAA Repair (1)^26^  EVAR (1)^32^  LEB (1)^31^ | ⨁⨁◯◯ Low | p = 0.72, I² = 0%;  Sensitivity analysis: RR 0.97 (95% CI 0.78-1.21), p = 0.77 |
| **Acute Kidney Injury (AKI)** | 23 per 1 000 | **46 per 1 000** (35 to 61) | **RR 1.98** (1.49 to 2.63) | 6 188 (2 studies)^25,31^ | Open AAA Repair (1)^25^  LEB (1)^31^ | ⨁⨁⨁◯ Moderate | p < 0.001, I² = 0%;  Not enough data for sensitivity analysis |
| **Renal Failure Requiring Dialysis** | 16 per 1 000 | **28 per 1 000** (18 to 44) | **RR 1.76** (1.13 to 2.74) | 15 017 (5 studies)^21,22,26,30,31^ | Open AAA Repair (3)^21,22,26^  EVAR (1)^30^  LEB (1)^31^ | ⨁⨁◯◯ Low | p = 0.010, I² = 40%;  Sensitivity analysis: RR 1.90 (95% CI 1.14-3.16), p = 0.010 |
| ICU vs General Ward | 16 per 1 000 | **30 per 1 000** (18 to 49) | **RR 1.90** (1.14 to 3.16) | 14 913 (4 studies)^21,22,30,31^ | Open AAA Repair (2)^21,22^  EVAR (1)^30^  LEB (1)^31^ | ⨁⨁◯◯ Low | p = 0.010, I² = 49%;  Not enough data for sensitivity analysis |
| Open AAA Repair | 12 per 1 000 | **60 per 1 000** (6 to 594) | **RR 4.84** (0.49 to 47.96) | 648 (3 studies)^21,22,26^ |  | ⨁◯◯◯ Very low | p = 0.18, I² = 66%;  Sensitivity analysis: RR 15.85 (95% CI 2.03-123.89), p = 0.008 |
| **Respiratory Complications** | 13 per 1 000 | **15 per 1 000** (8 to 28) | **RR 1.12** (0.60 to 2.11) | 36 213 (4 studies)^21,25,26,32^ | Open AAA Repair (3)^21,25,26^  EVAR (1)^32^ | ⨁◯◯◯ Very low | p = 0.72, I² = 83%;  Sensitivity analysis: RR 1.41 (95% CI 0.72-2.73), p = 0.31 |
| ICU vs Intermediary Level of Care | 246 per 1 000 | **143 per 1 000** (79 to 256) | **RR 0.58** (0.32 to 1.04) | 282 (2 studies)^25,26^ | Open AAA Repair (2)^25,26^ | ⨁◯◯◯ Very low | p = 0.40, I² = Not applicable;  Not enough data for sensitivity analysis |
| Open AAA Repair | 243 per 1 000 | **247 per 1 000** (78 to 791) | **RR 1.02** (0.32 to 3.26) | 596 (3 studies)^21,25,26^ |  | ⨁◯◯◯ Very low | p = 0.97, I² = 88%;  Sensitivity analysis: RR 1.42 (95% CI 0.37-5.48), p = 0.61 |
| **Respiratory Failure Requiring Mechanical Ventilation** | 13 per 1 000 | **36 per 1 000** (13 to 101) | **RR 2.83** (1.00 to 8.01) | 9 185 (5 studies)^21,22,25,26,30^ | Open AAA Repair (4)^21,22,25,26^  EVAR (1)^30^ | ⨁◯◯◯ Very low | p = 0.050, I² = 67%;  Sensitivity analysis: RR 4.25 (95% CI 1.61-11.18), p = 0.003 |
| ICU vs General Ward | 11 per 1 000 | **60 per 1 000** (19 to 192) | **RR 5.50** (1.73 to 17.54) | 8 903 (3 studies)^21,22,30^ | Open AAA Repair (2)^21,22^  EVAR (1)^30^ | ⨁⨁◯◯ Low | p = 0.004, I² = 66%;  Not enough data for sensitivity analysis |
| ICU vs Intermediary Level of Care | 45 per 1 000 | **28 per 1 000** (8 to 102) | **RR 0.61** (0.17 to 2.25) | 282 (2 studies)^25,26^ | Open AAA Repair (2)^25,26^ | ⨁◯◯◯ Very low | p = 0.46, I² = 0%;  Not enough data for sensitivity analysis |
| Open AAA Repair | 27 per 1 000 | **74 per 1 000** (13 to 431) | **RR 2.69** (0.46 to 15.76) | 826 (4 studies)^21,22,25,26^ |  | ⨁◯◯◯ Very low | p = 0.27, I² = 76%;  Sensitivity analysis: RR 5.61 (95% CI 1.26-24.86), p = 0.020 |
| **Infection** | 46 per 1 000 | **49 per 1 000** (32 to 76) | **RR 1.08** (0.70 to 1.67) | 8 955 (4 studies)^21,25,26,30^ | Open AAA Repair (3)^21,25,26^  EVAR (1)^30^ | ⨁◯◯◯ Very low | p = 0.71, I² = 58%;  Sensitivity analysis: RR 1.11 (95% CI 0.60–2.08), p = 0.74 |
| ICU vs General Ward | 29 per 1 000 | **40 per 1 000** (31 to 50) | **RR 1.37** (1.08 to 1.72) | 8 673 (2 studies)^21,30^ | Open AAA Repair (1)^21^  EVAR (1)^30^ | ⨁⨁⨁◯ Moderate | p = 0.008, I² = 0%;  Not enough data for sensitivity analysis |
| ICU vs Intermediary Level of Care | 347 per 1 000 | **260 per 1 000** (146 to 461) | **RR 0.75** (0.42 to 1.33) | 282 (2 studies)^25,26^ | Open AAA Repair (2)^25,26^ | ⨁◯◯◯ Very low | p = 0.32, I² = 35%;  Not enough data for sensitivity analysis |
| Open AAA Repair | 276 per 1 000 | **262 per 1 000** (138 to 503) | **RR 0.95** (0.50 to 1.82) | 596 (3 studies)^21,25,26^ |  | ⨁◯◯◯ Very low | p = 0.88, I² = 58%;  Sensitivity analysis: RR 1.24 (95% CI 0.63-2.46), p = 0.53 |
| **Surgical Site Infection** | 3 per 1 000 | **2 per 1 000** (1 to 4) | **RR 0.75** (0.45 to 1.23) | 41 909 (4 studies)^25,26,31,32^ | Open AAA Repair (2)^25,26^  EVAR (1)^32^  LEB (1)^31^ | ⨁⨁◯◯ Low | p = 0.25, I² = 0%;  Sensitivity analysis: RR 0.74 (95% CI 0.45-1.24), p = 0.25 |
| ICU vs Intermediary Level of Care | 40 per 1 000 | **33 per 1 000** (6 to 173) | **RR 0.83** (0.16 to 4.30) | 282 (2 studies)^25,26^ | Open AAA Repair (2)^25,26^ | ⨁◯◯◯ Very low | p = 0.83, I² = 0%;  Not enough data for sensitivity analysis |
| Open AAA Repair | 40 per 1 000 | **33 per 1 000** (6 to 173) | **RR 0.83** (0.16 to 4.30) | 282 (2 studies)^25,26^ |  | ⨁◯◯◯ Very low | p = 0.83, I² = 0%;  Not enough data for sensitivity analysis |
| **Re-Admission** | 93 per 1 000 | **180 per 1 000** (112 to 290) | **RR 1.93** (1.20 to 3.12) | 2 819 (2 studies)^21,27^ | Any Vascular Procedure (1)^27^  Open AAA Repair (1)^21^ | ⨁⨁⨁◯ Moderate | p = 0.007, I² = 0%;  Not enough data for sensitivity analysis |
| **30-Day or In-Hospital Major Bleeding** | 20 per 1 000 | **28 per 1 000** (21 to 37) | **RR 1.37** (1.03 to 1.81) | 9 059 (5 studies)^21,24-26,30^ | Open AAA Repair (3)^21,25,26^  EVAR (1)^30^  CEA (1)^24^ | ⨁⨁⨁◯ Moderate | p = 0.030, I² = 0%;  Sensitivity analysis: RR 1.38 (95% CI 1.04-1.84), p = 0.030 |
| ICU vs General Ward | 19 per 1 000 | **25 per 1 000** (19 to 34) | **RR 1.36** (1.01 to 1.82) | 8 673 (2 studies)^21,30^ | Open AAA Repair (1)^21^  EVAR (1)^30^ | ⨁⨁⨁◯ Moderate | p = 0.040, I² = 0%;  Not enough data for sensitivity analysis |
| ICU vs Intermediary Level of Care | 46 per 1 000 | **70 per 1 000** (24 to 203) | **RR 1.54** (0.53 to 4.45) | 386 (3 studies)^24-26^ | Open AAA Repair (2)^25,26^  CEA (1)^24^ | ⨁◯◯◯ Very low | p = 0.43, I² = 0%;  Not enough data for sensitivity analysis |
| Open AAA Repair | 37 per 1 000 | **74 per 1 000** (26 to 210) | **RR 1.98** (0.69 to 5.64) | 596 (3 studies)^21,25,26^ |  | ⨁◯◯◯ Very low | p = 0.20, I² = 0%;  Sensitivity analysis: RR 2.30 (95% CI 0.74-7.14), p = 0.15 |
| **Re-Intervention Within Index Admission** | 16 per 1 000 | **20 per 1 000** (14 to 31) | **RR 1.24** (0.83 to 1.87) | 42 453 (6 studies)^21,22,24,25,31,32^ | Open AAA Repair (3)^21,22,25^  EVAR (1)^32^  CEA (1)^24^  LEB (1)^31^ | ⨁◯◯◯ Very low | p = 0.30, I² = 50%;  Sensitivity analysis: RR 1.28 (95% CI 0.82-1.99), p = 0.27 |
| ICU vs General Ward | 29 per 1 000 | **28 per 1 000** (19 to 41) | **RR 0.95** (0.64 to 1.41) | 6 554 (3 studies)^21,22,31^ | Open AAA Repair (2)^21,22^  LEB (1)^31^ | ⨁⨁◯◯ Low | p = 0.80, I² = 2%;  Not enough data for sensitivity analysis |
| ICU vs Intermediary Level of Care | 12 per 1 000 | **42 per 1 000** (4 to 445) | **RR 3.64** (0.35 to 38.30) | 282 (2 studies)^24,25^ | Open AAA Repair (1)^25^  CEA (1)^24^ | ⨁◯◯◯ Very low | p = 0.28, I² = 46%;  Not enough data for sensitivity analysis |
| Open AAA Repair | 7 per 1 000 | **45 per 1 000** (12 to 173) | **RR 6.45** (1.68 to 24.69) | 722 (3 studies)^21,22,25^ |  | ⨁⨁⨁◯ Moderate | p = 0.007, I² = 0%;  Not enough data for sensitivity analysis |
| **Length of Hospital Stay** |  | MD **0.59 higher** (0.53 lower to 1.71 higher) | - | 42 105 (5 studies)^20,24,25,31,32^ | Open AAA Repair (1)^25^  EVAR (1)^32^  CEA (2)^20,24^  LEB (1)^31^ | ⨁◯◯◯ Very low | p = 0.30, I² = 100%;  Sensitivity analysis: MD 0.10 (95% CI -0.10-0.30), p = 0.33 |
| ICU vs General Ward |  | MD **0.9 higher** (0.86 lower to 2.66 higher) | - | 6 206 (2 studies)^20,31^ | CEA (1)^20^  LEB (1)^31^ | ⨁◯◯◯ Very low | p = 0.32, I² = Not applicable;  Not enough data for sensitivity analysis |
| ICU vs Intermediary Level of Care |  | MD **0.72 higher** (2.93 lower to 4.37 higher) | - | 282 (2 studies)^24,25^ | Open AAA Repair (1)^25^  CEA (1)^24^ | ⨁◯◯◯ Very low | p = 0.70, I² = Not applicable;  Not enough data for sensitivity analysis |
| Carotid Endarterectomy |  | MD **0.89 higher** (1.18 lower to 2.97 higher) | - | 300 (2 studies)^20,24^ |  | ⨁◯◯◯ Very low | p = 0.40, I² = Not applicable;  Not enough data for sensitivity analysis |
| ***The risk in the intervention group** (and its 95% confidence interval) is based on the assumed risk in the comparison group and the **relative effect** of the intervention (and its 95% CI).  **CI:** confidence interval; **MD:** mean difference; **OR:** odds ratio; **RR:** risk ratio; **ICU:** intensive care unit; **EVAR:** Endovascular Aneurysm Repair; **AAA:** Abdominal Aortic Aneurysm; **CEA:** Carotid Endarterectomy; **LEB:** Lower Extremity Bypass. | | | | | | | |
| **GRADE Working Group grades of evidence** **High certainty:** we are very confident that the true effect lies close to that of the estimate of the effect. **Moderate certainty:** we are moderately confident in the effect estimate: the true effect is likely to be close to the estimate of the effect, but there is a possibility that it is substantially different. **Low certainty:** our confidence in the effect estimate is limited: the true effect may be substantially different from the estimate of the effect. **Very low certainty:** we have very little confidence in the effect estimate: the true effect is likely to be substantially different from the estimate of effect. | | | | | | | |

| **Supplementary Table S4.** Full details of factors influencing admission to intensive care of included observational studies comparing the impact of intensive care admission to other levels of care for patients after elective or emergency vascular surgery. ICU = intensive care unit; AAA = abdominal aortic aneurysm; NYHA = New York Heart Association; POSSUM = Physiological and Operative Severity Score for the enumeration of Mortality and morbidity; ASA = American Society of Anaesthesiologists; IQR = Interquartile Range. | | | |
| --- | --- | --- | --- |
| **First Author** | **Type of Analysis** | **Factors Significantly Influencing ICU Admission** | **Non-Significant Factors** |
| D. Bertges^21^ | Unadjusted Univariate Analysis | Less frequent use of supra-renal clamps (4.9% vs 15.7%, p < 0.005)  Less frequent use of epidural catheters (46.8% vs 69.6%, p = 0.021)  Higher use of pulmonary artery catheters (67.3% vs 52.2%, p < 0.001)  Higher use of red blood cells transfusion (0.8 vs 0.1 units, p < 0.001)  Longer operating time (4:41 hours vs 4:08 hours, p < 0.001)  Longer procedural time (3:30 hours vs 2:50 hours, p < 0.001)  Higher volume of intravenous crystalloid/colloid infusions (4310 vs 3546 mL, p < 0.001) | Infra-renal clamps  Juxta-renal clamps  Tube Prosthetic Graft  Bifurcated Prosthetic Graft  Re-implantation of Inferior Mesenteric Artery  Inflammatory Abdominal Aortic Aneurysm  Urine output  Estimated blood loss  Cell saver transfusions |
| D. Angel^24^ | Unadjusted Univariate Analysis | Increasing age (p = 0.023) | Sex |
| C. Callaghan^25^ | Unadjusted Univariate Analysis | History of angina (42% vs 15%, p < 0.01)  History of myocardial infarction (31% vs 14%, p = 0.05)  Worse left ventricular function on echocardiography, good:moderate:poor (9:7:3 vs 30:6:1, p < 0.01)  Higher NYHA score, 1:2:3:4 (11:11:4:0 vs 99:46:3:0, p < 0.01)  Increasing AAA diameter (median 6.9 (IQR 6.3-7.6) vs 6.0 (5.7-7.0), p = 0.01)  Increasing POSSUM score (median 21.5 (IQR 18-27) vs 18 (16-22), p < 0.01)  Higher ASA score, 1:2:3:4 (0:5:18:2 vs 3:67:72:1, p = 0.01)  Cases not performed by trainees (8% admitted to ICU in operations performed by trainee vs 34% admitted to other level of care in operation performed by trainee, p < 0.01)  Increasing operative time, mins (median 205 (IQR 160-249) VS 175 (148-207), p = 0.02)  Higher intra-operative blood loss, ml (median 2500 (IQR 1200-4500) vs 1500 (850-2200) | Median age  Male Sex  Regular smoker within 6 months of operation  History of diabetes  History of hypertension  Renal impairment  Median POSSUM operative severity score |
| M.Cleary^26^ | Unadjusted Univariate Analysis | Higher physiological POSSUM (pPOSSUM) scores (19.2 +/- 0.7 vs 17.5 +/- 0.5, p = 0.048)  Higher total POSSUM (tPOSSUM) scores (35.5 +/- 0.8 vs 33.2 +/- 0.5, p = 0.02) |  |
| C. Hicks^30^ | Multivariable Logistic Regression | Nonwhite race (15.1% vs 11.0%, p < 0.001) Increasing Charlson comorbidity index (p < 0.001)  Smoking status (p < 0.001)  Congestive heart failure (7.8% vs 5.7%, p < 0.001)  Coronary artery disease (46.7% vs 42.4%, p < 0.001)  Chronic obstructive pulmonary disease (31.7% vs 26.7%, p < 0.001)  Diabetes (20.3% vs 18.3%, p = 0.02)  Hypertension (79.4% vs 76.2%, p < 0.001)  Physician specialty (p < 0.001)  Increasing operating room time, hrs (median 3.0 (IQR 2.5-3.8) vs 2.8 (2.3-3.5), p < 0.001)  Need for intraoperative transfusion (1.4% vs 0.5%, p < 0.001) Urban hospital setting (91.0% vs 86.1%, p < 0.001) Non-teaching hospital setting (38.4% admitted to ICU at teaching hospital vs 51.7%, p < 0.001) Geographic region (p < 0.001)  Increasing hospital volume (p < 0.001) | Median age  Insurance status  End-stage renal disease  Hospital bed size |
| H. Alshaikh^31^ | Multivariable Logistic Regression | Coronary artery disease (47.2% vs 40.9%, p < 0.001)  History of myocardial infarction (14.6% vs 12.0%, p = 0.02) Diabetes mellitus (36.9% vs 33.0%, p = 0.002)  Chronic obstructive pulmonary disease (26.9% vs 24.5%, p = 0.04)  Operating room time, hrs (median 3.5 (IQR 2.8-4.5) vs 3.2 (2.5-4.1), p < 0.001)  Rural hospital (12.7% vs 10.8%, p = 0.02)  Non-teaching hospital (66.0% vs 48.9%, p < 0.01)  Small bed capacity (p < 0.001)  Geographic region (p < 0.001)  Perioperative transfusion (2.0% vs 1.3%, p = 0.04) | Age  Sex  Race  Insurance status  Tobacco use  Congestive heart failure  Hypertension  Hyperlipidaemia  Chronic kidney disease  End-stage renal disease |
| T.Cheng^32^ | Unadjusted Univariate Analysis | Non-male sex (80.5% male vs 81.8%, p < 0.024) Non-white race (90.5% white vs 92.3%, p < 0.001) Insurance type (p = 0.017) Smoking status (p = 0.049) Absence of congestive heart failure (10.5% vs 11.9%, p = 0.002) No pre-operative aspirin (65.3% pre-op aspirin vs 67.4%, p = 0.003) Use of P2Y12 antagonist (13.9% vs 12.8%, p = 0.029) Smaller AAA diameter (54.2 +/- 10.8 vs 55.1 +/- 10.8mm, p < 0.001) Use of general anaesthetic (94.2% vs 91.1%, p < 0.001) Increased contrast use, ml (103 +/- 61.7 vs 95.4 +/- 57.8, p < 0.001)  Increased transfusion units (0.2 +/- 1.1 vs 0.2 +/- 1 unit, p < 0.001) Not extubated in operating room (92.2% extubated in operating room vs 97.1%, p < 0.001) Increased operating time, min (132.4 +/- 69.2 vs 130.3 +/- 68.1, p = 0.037) | Mean age  Pre-operative living status  Hypertension  Obesity  Coronary artery disease  Chronic obstructive pulmonary disease  Diabetes  Previous AAA repair  Dialysis dependence  Previous percutaneous coronary intervention  Previous coronary bypass graft  Previous lower extremity revascularisation  Statin use  Anticoagulant use  Concurrent iliac aneurysm  Estimated blood loss during procedure |
